# Wöchentliche versus dreiwöchentliche Cisplatingabe bei der postoperativen Radiochemotherapie von lokal fortgeschrittenen Kopf-Hals-Plattenepithelkarzinomen – Ergebnisse der JCOG1008-Studie

**DOI:** 10.1007/s00066-022-01970-x

**Published:** 2022-06-20

**Authors:** Alexander Rühle, Nils H. Nicolay

**Affiliations:** 1 Klinik für Strahlenheilkunde, Universitätsklinikum Freiburg, Robert-Koch-Str. 3, 79106 Freiburg, Deutschland; 2 Konsortium für Translationale Krebsforschung (DKTK), Partnerstandort Freiburg, Deutsches Krebsforschungszentrum (DKFZ), Heidelberg, Deutschland

## Hintergrund und Ziel der Studie

Sowohl in der definitiven als auch in der postoperativen Situation stellt die dreiwöchentliche Administration von 100 mg/m^2^ Cisplatin aufgrund einer Vielzahl prospektiver Phase-III-Studien den Goldstandard bei einer Radiochemotherapie (RCT) lokoregionär fortgeschrittener Kopf-Hals-Plattenepithelkarzinome (LA-HNSCC) dar. In der klinischen Praxis wird aufgrund der besseren Steuerbarkeit und der geringeren Akuttoxizität oftmals auf das wöchentliche Schema mit 30–40 mg/m^2^ Cisplatin zurückgegriffen, obgleich die Evidenz für eine onkologische Äquivalenz beider Schemata strittig ist. Die JCOG1008-Studie, eine multizentrische Phase-II/III-Studie, prüfte daher die Nichtunterlegenheit der wöchentlichen Gabe von 40 mg/m^2^ Cisplatin gegenüber dem etablierten dreiwöchentlichen Schema in der postoperativen Situation.

## Patienten und Methoden

LA-HNSCC-Patienten mit postoperativen Hochrisikoeigenschaften (Lymphknotenmetastasen mit Kapseldurchbruch und/oder positive Resektionsränder) wurden zwischen dreiwöchentlichen Cisplatininfusionen mit 100 mg/m^2^ und wöchentlichen mit 40 mg/m^2^ parallel zur postoperativen Radiotherapie (RT, 66 Gy in 33 Fraktionen) randomisiert. Der primäre Endpunkt der Phase-II-Studie war die Komplettierungsrate der Chemotherapie (definiert als kumulative Cisplatindosis von mindestens 200 mg/m^2^), während der primäre Endpunkt der anschließenden Phase-III-Studie das Gesamtüberleben (OS) war. Eine Nichtunterlegenheit des wöchentlichen Schemas wurde angenommen, wenn die obere Grenze des Konfidenzintervalls für die Hazard Ratio (HR) des OS den Wert von 1,32 nicht überschritt.

## Ergebnisse

Insgesamt 261 Patienten wurden zwischen Oktober 2012 und Dezember 2018 in die Studie eingeschlossen und zwischen dem dreiwöchentlichen (*n* = 132) und dem wöchentlichen Schema (*n* = 129) randomisiert. Die Komplettierungsrate der Chemotherapie (≥ 200 mg/m^2^ Cisplatin) betrug 86,8 % mit dem wöchentlichen und 93,2 % mit dem dreiwöchentlichen Schema. Die mediane kumulativ applizierte Cisplatindosis lag bei 239 mg/m^2^ im wöchentlichen und bei 280 mg/m^2^ im dreiwöchentlichen Arm. Mit einer HR von 0,69 (99,1 %-Konfidenzintervall: 0,374–1,273) für das OS zugunsten des wöchentlichen Schemas konnte die Nichtunterlegenheit der wöchentlichen Cisplatintherapie gezeigt werden (einseitiger *p*-Wert für die Nichtunterlegenheit = 0,027). Nach einer medianen Nachbeobachtungszeit von 2,2 Jahren betrug das 3‑Jahres-Gesamtüberleben 71,6 % im wöchentlichen und 59,1 % im dreiwöchentlichen Cisplatinarm. Auch die HR für das rezidivfreie (0,71) und lokalrezidivfreie Überleben (0,73) zeigten einen Trend zugunsten des wöchentlichen Cisplatinschemas. Es ließ sich keine Subgruppe identifizieren, bei der die HR > 1 betrug und somit einen Vorteil für das dreiwöchentliche Cisplatinschema suggeriert hätte. Zudem ließen sich signifikant höhere Toxizitätsraten in der Gruppe mit dreiwöchentlicher Cisplatinbehandlung (Neutropenie Grad ≥ 3: 49 % versus 35 %, Infektion Grad ≥ 3: 12 % versus 7 %, Nephrotoxizität Grad ≥ 1: 40 % versus 30 %, Tinnitus Grad ≥ 1: 25 % versus 5 %, akute Grad-≥ 4-Toxizitäten: 19 % versus 8 %) feststellen.

## Schlussfolgerung der Autoren

Wöchentliche Cisplatingaben mit 40 mg/m^2^ sind in der postoperativen RCT bei LA-HNSCC dem dreiwöchentlichen 100 mg/m^2^-Schema onkologisch nicht unterlegen und weisen geringere Toxizitätsraten auf. Ein solches Schema ist in der postoperativen Situation daher eine gute Alternative zum bisherigen Therapiestandard mit dreiwöchentlichen Gaben von Cisplatin 100 mg/m^2^.

## Kommentar

Die JCOG1008-Studie hatte zum Ziel, die in der klinischen Routine häufig diskutierte und in vielen retrospektiven und wenigen prospektiven Studien untersuchte Fragestellung zu klären, ob ein wöchentliches Cisplatinschema mit 40 mg/m^2^ gegenüber dem auf den EORTC-22931- und RTOG-9501-Studien basierenden Therapiestandard mit dreiwöchentlich Cisplatin 100 mg/m^2^ onkologisch gleichwertig ist [1–3]. In der definitiven Situation bei LA-HNSCC hat die RTOG-91-11-Studie Cisplatin 100 mg/m^2^ in Woche 1, 4 und 7 als Standard etabliert [4]. Die beiden Landmark-Studien zur postoperativen RCT bei histopathologischen Hochrisikoeigenschaften, die EORTC-22931- und RTOG-9501-Studie, begründeten das dreiwöchentliche Cisplatinschema dann auch in der postoperativen Situation [2, 3]. Zumindest bei Plattenepithelkarzinomen anderer Lokalisation, wie beispielsweise der Zervix, sind jedoch wöchentliche 40 mg/m^2^-Applikationen basierend auf großen randomisierten Studien die etablierten Schemata [5].

In einer systematischen Übersichtsarbeit, die 4209 HNSCC-Patienten aus 52 prospektiven Studien einschloss, konnten Szturz et al. keine Unterschiede im OS zwischen wöchentlichen und dreiwöchentlichen Schemata detektieren, wohingegen das wöchentliche Schema zumindest im definitiven Setting mit verminderten Toxizitäten einherging [6]. Allerdings hatte nur eine der berücksichtigten Studien eine Randomisierung zwischen dem wöchentlichen und dreiwöchentlichen Schema durchgeführt [7]. In einer Studie von Bauml und Kollegen, bei der Daten von 2901 Patienten der amerikanischen *Veterans-Affairs*-Kohorte nach definitiver RCT analysiert wurden, konnte nach *Propensity Score Matching* kein Unterschied zwischen dem wöchentlichen und dreiwöchentlichen Schema im Hinblick auf das OS festgestellt werden. Jedoch zeigten sich auch hier signifikant mehr Toxizitäten im dreiwöchentlichen Cisplatinarm [8].

In einer der hier diskutierten JCOG1008-Studie ähnlichen randomisierten indischen Phase-III-Studie verglichen Noronha und Kollegen eine konkomitante Therapie mit 30 mg/m^2^ Cisplatin wöchentlich mit 100 mg/m^2^ Cisplatin dreiwöchentlich: Hier war die lokoregionäre Kontrolle nach 2 Jahren als primärer Endpunkt der Studie in der wöchentlichen Cisplatingruppe signifikant geringer (58,5 % versus 73,1 %, HR = 1,76, *p* = 0,014; [9]). Die hier diskutierte JCOG1008-Studie hebt sich allerdings methodisch von der vorherigen Studie ab: In der Studie von Noronha und Kollegen wurden beispielsweise noch veraltete Bestrahlungstechniken (z. B. Telekobaltbestrahlung mit Gegenfeldern) verwendet und sowohl bei definitiv als auch postoperativ behandelten Patienten eingesetzt. Ein wesentlicher Unterschied zwischen beiden Studien war außerdem die in der JCOG1008-Studie verwendete höhere kumulative Cisplatindosis: Während in der japanischen Studie median 239 mg/m^2^ (Interquartilsrange [IQR] 199–277) Cisplatin im wöchentlichen Arm appliziert wurden, waren es nur 210 mg/m^2^ (IQR 180–210) in der indischen Studie. Angesichts des in zahlreichen retrospektiven Arbeiten demonstrierten Zusammenhangs zwischen der kumulativen Cisplatindosis und der lokoregionären Tumorkontrolle sowie der Wichtigkeit einer kumulativen Dosis von mindestens 200 mg/m^2^ scheinen also 30 mg/m^2^ wöchentlich keine ausreichende Dosis zu sein, wenn man Äquipotenz mit den dreiwöchentlichen Gaben von 100 mg/m^2^ erreichen will. Während in der JCOG1008-Studie 87 % der Patienten bei wöchentlicher Cisplatinadministration mindestens 200 mg/m^2^ erhielten, konnte bei wöchentlichen Gaben diese kumulative Dosis nur bei 58 % der Patienten in der indischen Studie erreicht werden, womit sich die unterschiedlichen Ergebnisse beider Studien zumindest partiell erklären lassen [10].

Unterschiede in den Patientencharakteristika sowie bei den Bestrahlungsparametern könnten weitere Gründe für die beobachteten Unterschiede zwischen diesen beiden Studien sein: Beispielsweise waren die Patienten in der indischen Studie im Durchschnitt 18 Jahre jünger (44 Jahre versus 62 Jahre im Median) und hatten deutlich häufiger Mundhöhlenkarzinome (87 % versus 46 %); ebenso unterschieden sich die median applizierten Dosen zwischen der indischen (60 Gy) und japanischen (66 Gy) Studie. Auch die Tatsache, dass das OS wie auch das rezidivfreie und krebsspezifische Überleben in der JCOG1008-Studie trotz der geringeren kumulativen Cisplatindosis im wöchentlichen Arm (median 239 mg/m^2^ versus 280 mg/m^2^) eher zugunsten der wöchentlichen Cisplatin-Kohorte ausfiel, unterstreicht die Wichtigkeit der kritischen Dosis von 200 mg/m^2^ und den fehlenden oder zumindest nur noch geringen Zusatznutzen bei höheren kumulativen Dosen [10].

Die geringeren Akuttoxizitätsraten im wöchentlichen Arm der JCOG1008-Studie (Grad ≥ 4: 8 % versus 19 %, *p* = 0,017) sind in Übereinstimmung mit den Ergebnissen von Noronha et al. (Grad-3–4-Toxizitäten: 72 % versus 85 %, *p* = 0,006). Der wöchentliche Arm der JCOG1008-Studie wies dabei in fast allen relevanten Domänen geringere Raten an höhergradigen Toxizitäten auf: 35 % versus 49 % für Neutropenie, 12 % versus 19 % für Dysphagie, 5 % versus 13 % für Nausea und 7 % versus 12 % für Infektionen. Konkordant mit den Daten von Noronha et al. zeigte sich auch ein signifikanter Vorteil zugunsten der wöchentlichen Cisplatinadministration im Hinblick auf die Häufigkeit eines Tinnitus (5 % versus 25 %). Wahrscheinlich sind die höheren Peak-Dosen bei Cisplatingaben von 100 mg/m^2^ die Ursache für die häufigere Ototoxizität im dreiwöchentlichen Schema. Inwieweit daher fraktionierte Hochdosisschemata, also beispielsweise 5 × 20 mg/m^2^ Cisplatin, im Hinblick auf onkologische Gleichwertigkeit und geringere Toxizität eine Alternative zu Cisplatin 100 mg/m^2^ an Tag 1, 22 und 43 sein könnten, bleibt eine relevante und bisher nicht suffizient beantwortete Frage.

Obgleich den Autoren zu der erfolgreichen Durchführung ihrer multizentrischen randomisierten Studie und dem erreichten hohen Evidenzgrad nur zu gratulieren ist, müssen doch einige wenige Unzulänglichkeiten benannt werden: Erstens ist die mediane Nachbeobachtungszeit von etwas mehr als zwei Jahren noch relativ kurz für eine gesicherte Aussage. Zweitens wird das Studienkollektiv dominiert von Mundhöhlenkarzinomen (46 %), wohingegen Larynxkarzinome (9 %) und Oropharynxkarzinome (14 %, davon 77 % p16-positiv) relativ selten vertreten sind, was die Übertragbarkeit der Ergebnisse auf diese Lokalisationen einschränkt. Drittens ist die mediane Zeitspanne von 7 Wochen zwischen Operation und Beginn der RCT bzw. 14 Wochen zwischen Operation und Ende der RCT länger, als es die aktuellen Leitlinien empfehlen.

In der definitiven Behandlungssituation wird die aktuell noch rekrutierende NRG-HN009-Studie, eine große multizentrische randomisierte Phase-II/III-Studie, hoffentlich die Frage beantworten, inwieweit die wöchentliche Cisplatingabe von 40 mg/m^2^ verglichen mit dreiwöchentlichen Cisplatingaben von 100 mg/m^2^ geringere Akuttoxizitäten verursacht und bezüglich des OS nicht unterlegen ist. Die randomisierte TROG12.01-Studie (Vergleich zwischen Cisplatin und Cetuximab bei HPV-positiven Oropharynxkarzinomen) verwendete beispielsweise bereits routinemäßig das wöchentliche Cisplatin-Schema mit 40 mg/m^2^.

Weiterhin offen bleibt zudem die Frage, inwieweit 2 Zyklen Cisplatin mit 100 mg/m^2^ onkologisch gleich effektiv zu bewerten sind wie 3 Zyklen. In der RTOG-0129-Studie, einer Phase-III-Studie zum Vergleich einer akzelerierten RCT mit 2 Zyklen Hochdosiscisplatin versus eine normofraktionierte RCT mit 3 Zyklen, wiesen Patienten mit lediglich einem Zyklus Cisplatin signifikant geringere OS-Raten auf, allerdings zeigte sich kein OS-Unterschied zwischen 2 und 3 Zyklen Hochdosiscisplatin [11]. Retrospektive multizentrische Daten deuten zwar auf eine Überlegenheit von kumulativen Dosen > 200 mg/m^2^ verglichen mit exakt 200 mg/m^2^ hin, allerdings nur bei HPV-negativen, nicht jedoch bei HPV-positiven LA-HNSCC-Patienten [12]. Lediglich 2 Zyklen Hochdosiscisplatin wurden in der RTOG-1016-Studie, einer randomisierten HPV-Deeskalationsstudie zum möglichen Ersatz von Cisplatin durch Cetuximab, appliziert – diese Studie präsentierte dabei überzeugende onkologische Daten für dieses Cisplatinschema (z. B. eine lokoregionäre Rezidivrate von lediglich noch 10 % nach 5 Jahren; [13]). In der De-ESCALaTE-HPV-Studie, welche dieselbe Fragestellung untersuchte wie die RTOG-1016-Studie, waren zwar 3 Zyklen Cisplatin vorgesehen, jedoch erhielt die Mehrheit der HPV-positiven Oropharynxkarzinompatienten nur 2 Zyklen [14].

## Fazit

Die hier diskutierte Phase-II/III-Studie zur Frage der geeigneten Cisplatindosierung im Rahmen der postoperativen RCT bei der Hochrisikosituation von LA-HNSCC zeigt die Nichtunterlegenheit von wöchentlichen Cisplatininfusionen mit 40 mg/m^2^ im Vergleich zu dreiwöchentlichen Gaben von 100 mg/m^2^ Cisplatin. Diese Studienergebnisse liefern einen hohen Evidenzgrad für die bereits in der klinischen Routine und einigen prospektive Studien (z. B. in den HPV-Deeskalationsstudien ECOG3311 und PATHOS) angewandte Praxis der konkomitanten wöchentlichen Cisplatinadministration bei der postoperativen RCT.

Gerade bei älteren Patienten mit Komorbiditäten müssen für das klassische Cisplatinschema 100 mg/m^2^ an Tag 1, 22 und 43 kritisch mögliche minimale Vorteile hinsichtlich der lokoregionären Kontrolle mit dem höheren Risiko höhergradiger Toxizitäten (und dadurch gegebenenfalls auch häufigeren therapiebedingten Todesfällen) abgewogen werden [15]. Das wöchentliche Schema mit 40 mg/m^2^ stellt speziell für diese Patientenpopulation in der postoperativen Situation wegen der geringeren Akuttoxizität ein attraktives Behandlungskonzept dar. In der JCOG1008-Studie waren immerhin 36 % der Patienten im Alter von mindestens 65 Jahren.

*Alexander Rühle und Nils H. Nicolay, Freiburg*
